# TNF-α blockade impairs *in vitro* tuberculous granuloma formation and down modulate Th1, Th17 and Treg cytokines

**DOI:** 10.1371/journal.pone.0194430

**Published:** 2018-03-15

**Authors:** Djalma A. Alves da Silva, Marcos V. da Silva, Cleyson C. Oliveira Barros, Patrícia B. Dias Alexandre, Rodolfo P. Timóteo, Jonatas S. Catarino, Helioswilton Sales-Campos, Juliana R. Machado, Denise B. R. Rodrigues, Carlo J. Oliveira, Virmondes Rodrigues

**Affiliations:** 1 Laboratory of Immunology, Department of Biological Sciences, Triângulo Mineiro Federal University, Uberaba, Minas Gerais, Brazil; 2 Uberaba Municipal Department of Health, Uberaba, Minas Gerais, Brazil; 3 Institute of Tropical Pathology and Public Health, Federal University of Goiás, Goiania, Goiás, Brazil; 4 Laboratory of Biopathology and Molecular Biology, University of Uberaba, Uberaba, Minas Gerais, Brazil; Universita Cattolica del Sacro Cuore, ITALY

## Abstract

Tuberculosis (TB) is a granulomatous disease that has affected humanity for thousands of years. The production of cytokines, such as IFN-γ and TNF-α, is fundamental in the formation and maintenance of granulomas and in the control of the disease. Recently, the introduction of TNF-α-blocking monoclonal antibodies, such as Infliximab, has brought improvements in the treatment of patients with chronic inflammatory diseases, but this treatment also increases the risk of reactivation of latent tuberculosis. Our objective was to analyze, in an *in vitro* model, the influence of Infliximab on the granulomatous reactions and on the production of antigen-specific cytokines (TNF-α, IFN-γ, IL-12p40, IL-10 and IL-17) from beads sensitized with soluble Bacillus Calmette-Guérin (BCG) antigens cultured in the presence of peripheral blood mononuclear cells (PBMC) from TB patients. We evaluated 76 individuals, with tuberculosis active, treated and subjects with positive PPD. Granuloma formation was induced in the presence or absence of Infliximab for up to 10 days. The use of Infliximab in cultures significantly blocked TNF-α production (p <0.05), and led to significant changes in granuloma structure, *in vitro*, only in the treated TB group. On the other hand, there was a significant reduction in the levels of IFN-γ, IL-12p40, IL-10 and IL-17 after TNF-α blockade in the three experimental groups (p <0.05). Taken together, our results demonstrate that TNF-α blockade by Infliximab directly influenced the structure of granuloma only in the treated TB group, but negatively modulated the production of Th1, Th17 and regulatory T cytokines in the three groups analyzed.

## Introduction

Tuberculosis (TB) is an infectious and contagious disease that has affected the world society for thousands of years [1]. Currently it is estimated that one-third of the world population is infected with *Mycobacterium tuberculosis* [2] of these, 90–95% of cases remain symptomatic and non-communicable in a state called latent tuberculosis [3,4]. Since *M*. *tuberculosis* resides essentially in vacuoles within macrophages, the protective immune response against the *Mycobacterium* is dependent on the interaction between infected cells and CD4^+^ T cells [5,6]. This interaction occurs after the dissemination of *M*. *tuberculosis* to regional lymph nodes, where bacterial antigens are presented by dendritic cells, promoting the expansion of antigen-specific T cells [7].

*M*. *tuberculosis* infect macrophages and induce the formation of granulomas, which are composed of differentiated macrophages, lymphocytes and other cells of the immune system [8]. Granuloma is the structure that restricts the growth and spread of *M*. *tuberculosis*, and Th1 profile cytokines are classically associated with this structure [7]. TNF-α acts in synergy with IFN-γ in the stimulation of nitric oxide (NO) production by macrophages, besides being a key cytokine in the formation and maintenance of granulomas [9]. Changes in the immunological status of the individual, such as treatment with immunosuppressant drugs and HIV infection, potentiate the risk of reactivation of the disease [10,11].

Recently, the increase in the therapeutic indication of immunobiologicals agents as TNF-α inhibitors has brought a new risk factor for reactivation of latent tuberculosis in the last two decades. The use of Immunobiologicals drugs has revolutionized the treatment of chronic inflammatory diseases, especially in rheumatoid arthritis (RA), juvenile idiopathic arthritis (JIA) [11], as well in Inflammatory Bowel Diseases [12,13]. Rheumatic disease alone increases by 2 to 4 times the risk of TB [14] and by 30 times with the use of Infliximab, [15] thus characterizing the use of immunobiologics as a risk factor for reactivation of TB [16]. Factors that disturb this delicate balance between mycobacteria and the maintenance of granuloma inevitably increase the risk of reactivation of the disease [11]. The understanding of microorganism-host interaction in granulomas is of paramount importance for the understanding of mycobacterial infections, but access to human granulomas through biopsies is very limited. The use of *in vitro* models that simulate the granulomatous reaction is an important strategy for the identification of mediators that influence its formation and maintenance [17,18]. In the view that the anti-tuberculosis immune response is distinct in patients with the active infection compared to post-treatment subjects or those with positive PPD (Purified Protein Derivative) intradermal test, we evaluated, *in vitro*, in these 3 groups of patients the effects of the formation of granuloma and the levels of cytokines involved in the control of *M*. *tuberculosis* in the presence of Infliximab, through peripheral blood mononuclear cells of patients with active tuberculosis, after treatment and individuals with positive PPD.

## Methods

### Patients

Blood samples were collected from 32 patients diagnosed with Active Tuberculosis (Active TB), 27 patients with previous TB episode who completed the treatment successfully (Treated TB) and 17 positive PPD individuals (PPD+ Control) with negative diagnosis for active TB and without previous history of tuberculosis. Patients with pulmonary or extra pulmonary forms of active and/or treated TB were included. Patients with active and treated disease were selected from Basic Health Units and UFTM General Hospital (Uberaba, State of Minas Gerais, Brazil). PPD+ subjects were volunteers with no previous history of tuberculosis, with induration > 10 mm in intradermal test (Statens Serun Institut, Copenhagen, Denmark). Blood of patients with active TB was collected until the 21^st^ day after the start of treatment, in order to minimize its interference. In all cases, the diagnosis of TB was defined through clinical, radiographic and laboratory criteria, according to guidelines of the World Health Organization (WHO) [19,20]. As criteria for inclusion in this study were considered: subjects aging over than 18 years that have not received organ transplantation or are in use of immunosuppressant’s, HIV infection, have clinical disease or not. All individuals accepted to participate in this study, and after clarification, they signed a consent form. This study was approved by the ethics committee of the Federal University of the Triângulo Mineiro (UFTM) under the protocols number: 852 and 1475.

### Obtaining peripheral blood mononuclear cells

Peripheral blood mononuclear cells (PBMC) were separated by density gradient in Ficoll-Hypaque (GE Health Care, Uppsala, Sweden), centrifuged at 400XG for 30 minutes at 21°C. They were resuspended in RPMI 1640 (GE) medium containing 50mM Hepes (GIBCO, Grand Island, NY, USA), 5% inactivated fetal bovine serum (GIBCO), 2mM L-glutamine (GIBCO), 40μg/mL gentamicin (Neoquímica, Anápolis, State of Goiás, Brazil), 1 mL 2β-mercaptoethanol (Merck, Darmstadt, Germany), in a final concentration of 1x10^6^/mL. They were then cultured in 96-well plates (FALCON, San Jose, CA, USA) in the presence of conjugated and non-conjugated BCG Beads.

### Obtaining BCG antigens

*Mycobacterium bovis* samples (Bacillus Calmette-Guérin—BCG), Moreau strain (Fundação Ataulpho de Paiva, Rio de Janeiro, State of Rio de Janeiro, Brazil) were used for the extraction of *Mycobacterium* antigens. Mycobacteria were resuspended in 0.85% physiological solution, incubated in a water bath at 90°C for 30 minutes, according to the manufacturer's protocol, and then autoclaved for 30 minutes. Soon after, they were centrifuged at 10,000xG at 4°C, for 30 minutes. The protein portion of the supernatant was collected, filtered through a 0.22μm filter (MILLIPORE, Molsheim, France), aliquoted and stored at -20°C. An aliquot was collected for dosing the protein concentration by the Bradford method (Pierce, Rockford, IL), according to the manufacturers protocol.

### Conjugation of BCG antigen to polyacrylamide beads

The above antigenic preparation was conjugated to the polyacrylamide beads, (BIO-GEL^®^ P-4 GEL BIO-RAD cat# 150–450 Hercules, CA, USA). 10 grams of beads were sterilized by gamma radiation at the dose of 100 cGy (Centigrays) (LINEAR ACCELERATOR VARIAN CLINAC 600 C, SN:310 Palo Alto, CA, USA), and hydrated in 1000 mL distilled water. After washing in 0.5M carbonate/bicarbonate buffer, 200 mg beads were incubated in this buffer for four hours in a water bath at 63°C with slow and continuous stirring. Next, 40 mg BCG antigens were added in the presence of 100 mg EDAC {N-ethyl-N '- (3-dimethylaminopropyl) Carbodiimide, Hydrochloride} SC 219152,(Santa Cruz Biotechnology, Dallas, Texas, USA) in 100 mL of sterile distilled water pH 6.5 at 4°C for 18 hours under slow and continuous stirring. Subsequently, the beads were washed and stored in sterile phosphate-buffered saline (PBS). Concomitantly, negative control beads were subjected to the same processes in the absence of BCG antigens, thus obtaining naked beads. At the time of use, beads were washed three times with incomplete RPMI medium. The whole procedure was carried out under axenic conditions, with sterile solutions and materials.

### Granuloma *in vitro* model and treatment with anti-TNF-α monoclonal antibody

The *in vitro* granuloma formation assay was adapted according to the procedure described by Silva-Teixeira 1993, as described below [21].

Cultures for granuloma formation assay were performed on 96-well flat bottom culture plate (FALCON, San Jose, CA, USA) at the concentration of 2.5 x 10^5^ cells/well for 10 days, kept at 37°C, in humid air containing 5% CO_2_. The cultures were performed in the presence of medium, soluble antigen (BCG), non-conjugated beads, BCG-conjugated beads in the presence and absence of 40 μg/mL of anti-TNF-α monoclonal antibody (Remicade^®^ 100mg Infliximab Janssen Biologics BV, Leiden—Nederland and Schering-Plow (Brinny) Company, County Cork—Ireland.).

**Fig 1:**
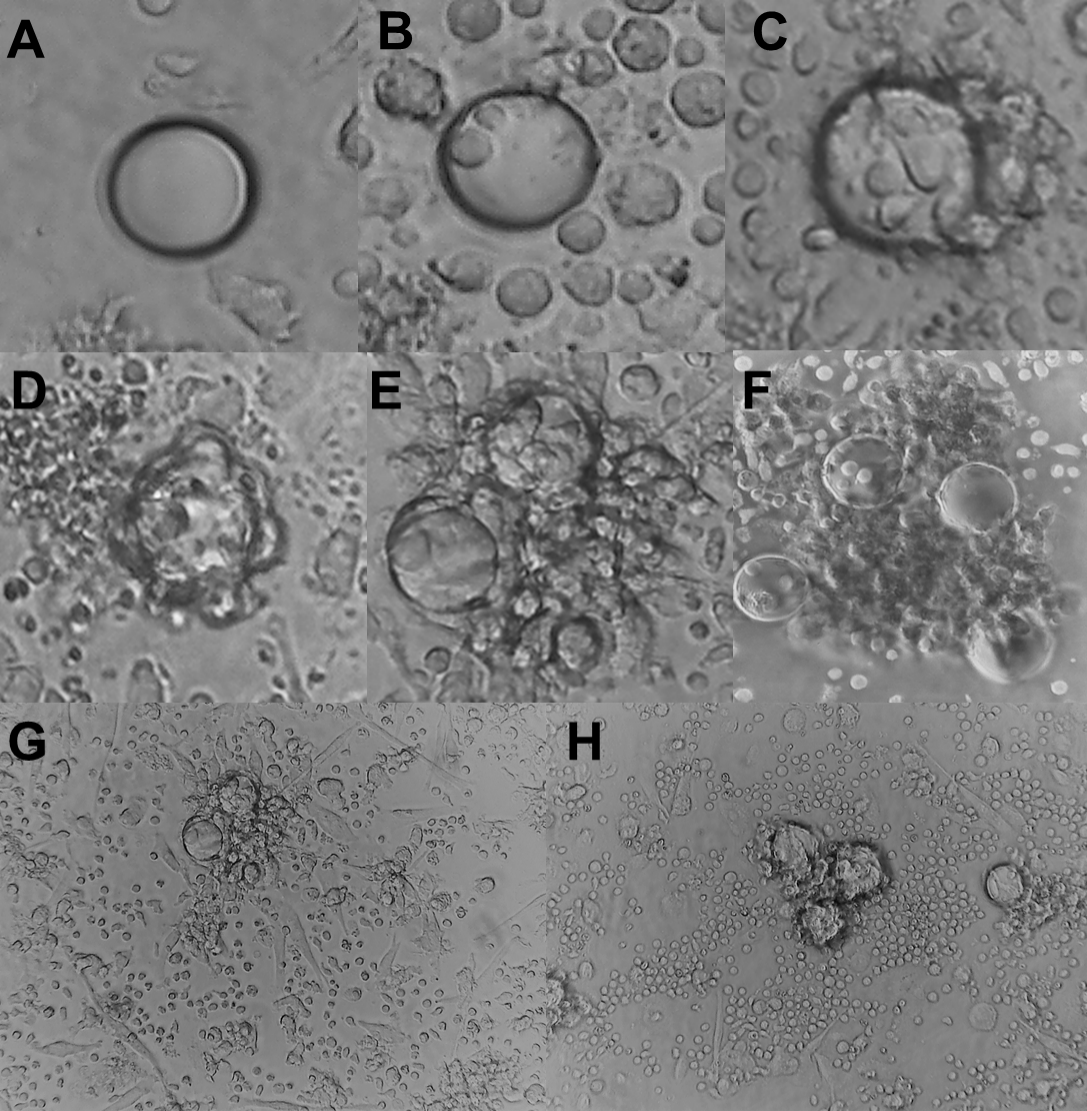
Classification of cellular reactivity around the beads, granuloma index. Representative image of cell reactivity index, in which A (No bead-bound cell), B (up to 5 bead-bound cells), C (5 or more bead-bound cells without cell migration or blast transformation), D (5 or more bead-bound cells, with cell migration and blast transformation), E (monolayer of cells covering the whole bead, cell migration and presence of blastic cells), and F (Multiple layers of bead-bound cells, cell migration and blast transformation). Representative image of an Treated TB patient at 10^th^ day without (G) or with Infliximabe.

The granuloma index was assessed by quantification of the level of cellular reactivity in around 50 beads/well, as described [22]. According to the formation of the granuloma, scores were assigned as shown in Fig 1: A) score 1 (No bead-bound cell); B) score 2 (up to 5 bead-bound cells), C) score 3 (5 or more bead-bound cells without cell migration or blastic transformation), D) score 4 (5 or more bead-bound cells with cell migration and blastic transformation); E) score 5 (monolayer of cells covering the whole bead, cell migration and presence of blastic cells); F) score 6 (multiple layers of bead-bound cells, cell migration and blast transformation). The results were expressed as the granuloma index calculated by the mean in each well.

### Quantification of cytokines in culture supernatants

The concentrations of TNF-α, IFN-γ, IL-10, IL-12p40 in the culture supernatants collected on the 5^th^ and 10^th^ day were measured by ELISA using monoclonal antibody pairs according to the manufacturer's specifications (BD Pharmingen, Franklin Lakes, NJ, USA) and IL-17 (R & D Systems, Minneapolis, MN, USA). Cytokine concentration was calculated using linear regression analysis of absorbance values obtained for recombinant cytokines, and was expressed in pg/mL. The sensitivity of the tests ranged from 2 to 20 pg/mL.

### Statistical analysis

Statistical analysis was performed using EXCEL 2007 for WINDOWS (MICROSOFT—USA), STATVIEW (ABACCUS-USA) and GRAPHPAD PRISM 5.0 (GRAPHPAD SOFTWARE—USA). The normal distribution of the quantitative variables was checked by the D-Agostino & Pearson test. Continuous variables that presented normal distribution were expressed as mean ± standard deviation and those with non-normal distribution were expressed in medians and percentiles. The variables that did not present normal distribution or did not have homogeneous variance were analyzed by the Mann-Whitney test for comparison of two independent groups or Kruskal-Wallis for three or more groups, with Dunn’s post-test when necessary. The analysis of the data corresponding to the repeated measurements was evaluated by the Wilcoxon test. Differences were considered statistically significant when p <0.05.

## Results

### Modulation of granuloma by TNF-α blockade

Here we evaluated the impact of TNF-α blockade by Infliximab on the ability of PBMCs derived from active and clinically cured tuberculosis patients and PPD+ healthy donors to form a granulomatous reaction through an *in vitro* model using mycobacterial antigens-coated polyacrylamide beads. In addition, we used this *in vitro* model of antigen-specific granulomatous reaction to evaluate the effects of TNF-α blockade on the ability of these PBMCs to produce cytokines related to the Th1, Th17 and T regulatory subsets.

**Fig 2:**
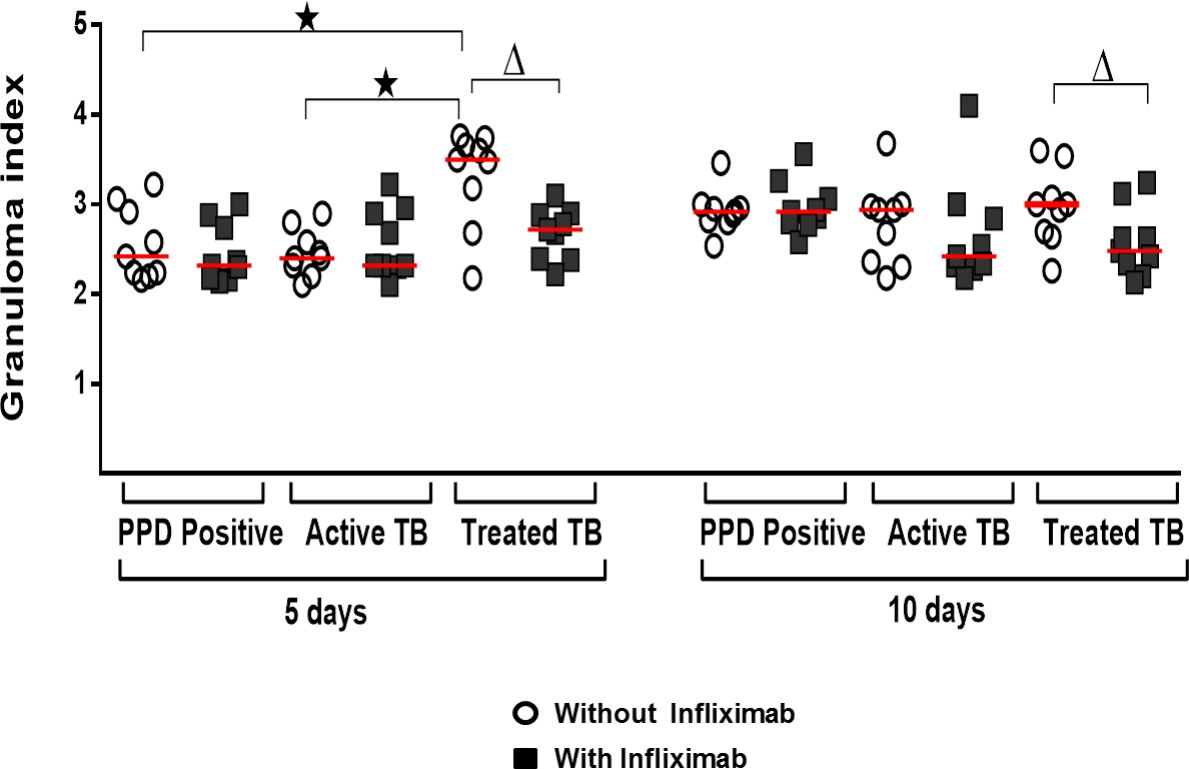
Effects of TNF-α blockade on in vitro granuloma formation. Granuloma index under different culture conditions–bead or BCG-bead—on days 5 and 10, in the presence or absence of infliximab. *significant difference between groups on the same day and in the same culture condition. Δ significant difference in the same group and same day of culture, after addition of Infliximab. Wilcoxon or Kruskal-Wallis tests followed by Dunn's post-test. Significant difference was p-value <0.05. The horizontal lines represent the medians, the bars represent the 25–75% percentiles and the vertical lines represent the 10–90% percentiles.

After the blockade of TNF-α by Infliximab, we sought to check for differences between the groups, and whether this blockade influenced or not the formation of the granuloma. On the 5^th^ and 10^th^ days, in order to evaluate the *in vitro* granuloma formation shown in Fig 1, the counts of 50 beads/well were expressed as means. On the 5^th^ day, in the absence of TNF-α blockade, the Treated TB group presented a significantly higher granuloma index than the PPD+ Control and Active TB groups (p = 0.01 and p = 0.005) (Kruskal-Wallis). On the 10^th^, no statistical differences were detected. TNFα blockade significantly reduced the granuloma index in the group with treated tuberculosis on the 5^th^ and 10^th^ day in the BCG-bead condition (p = 0.015 and p = 0.039) (Wilcoxon). In the other conditions, no significant difference was observed (Fig 2).

### Modulation of cytokine production by TNF-α blockade

**Fig 3:**
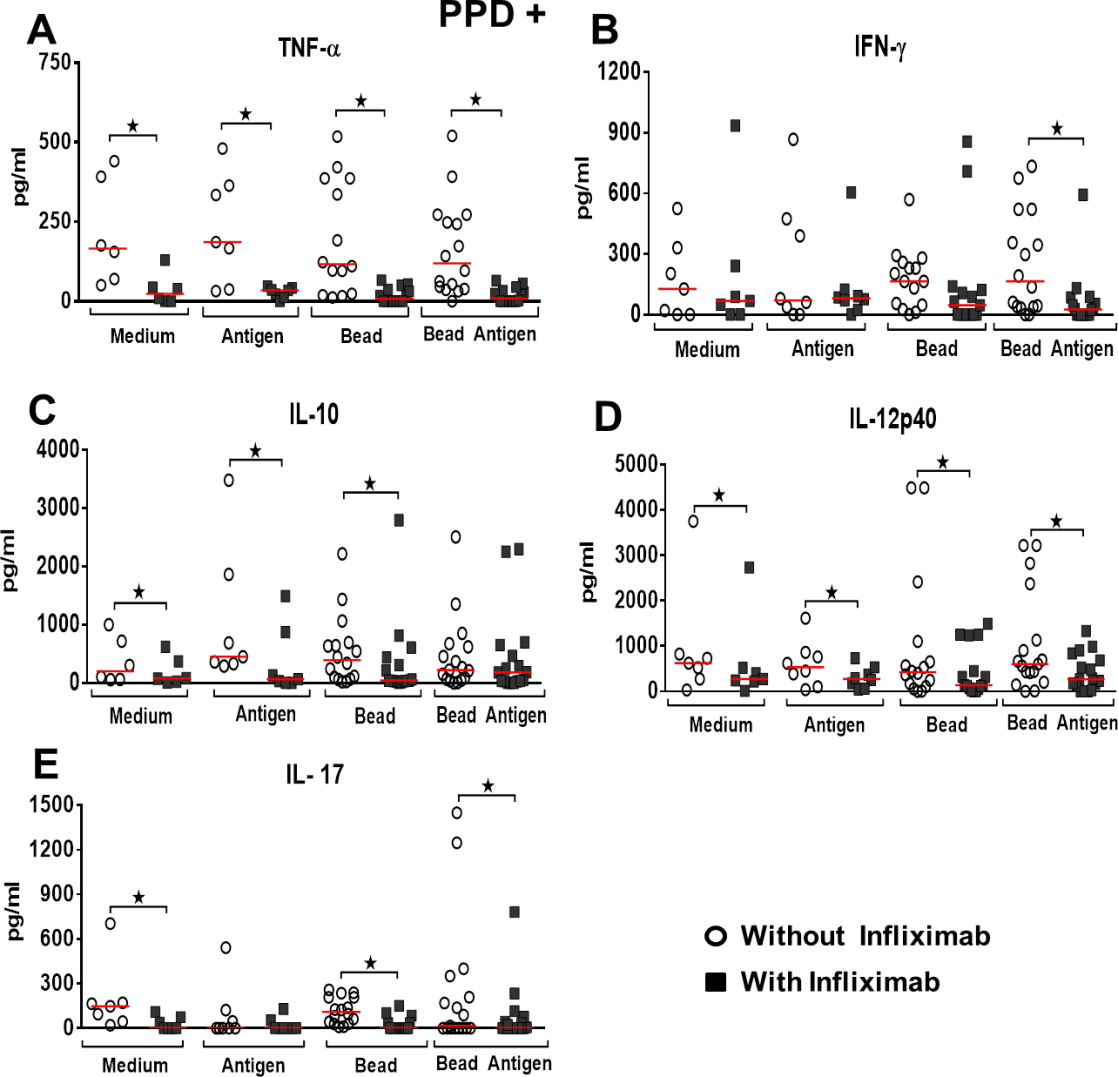
Effect of TNF-α blockade on cytokines production in PPD+ group. Levels of cytokines after granuloma formation, *in vitro*, PPD+ Control group (A-F) under different culture conditions—Medium or Antigen Medium and Bead or BCG-Bead, in the presence or absence of Infliximab. *Statistical differences between the conditions tested after the addition of Infliximab, in the different cytokines. Wilcoxon and Kruskal-Wallis tests, p-value <0.05. The horizontal lines represent the medians, the bars represent the 25–75% percentiles and the vertical lines represent the 10–90% percentiles.

**Fig 4:**
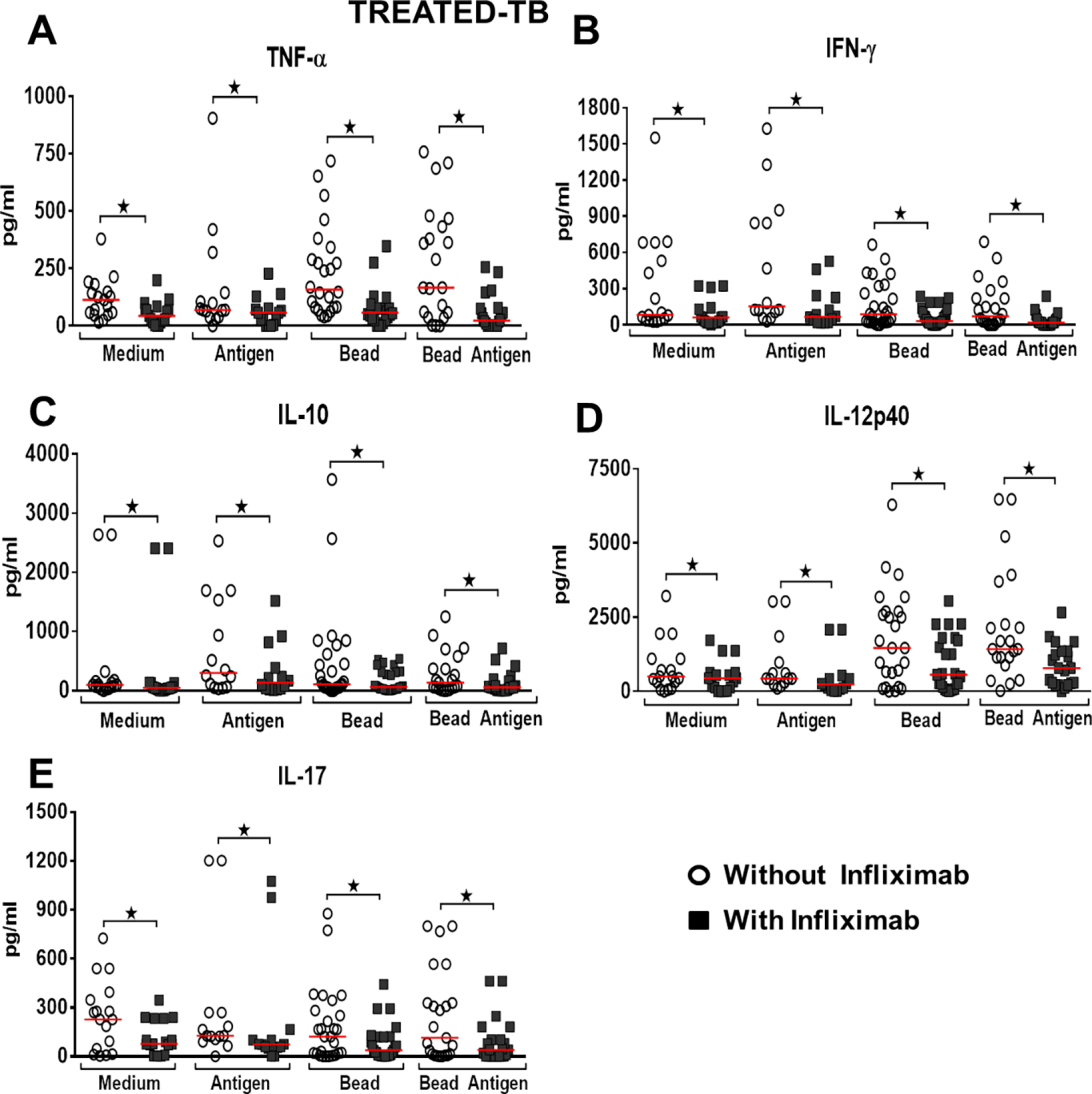
Effect of TNF-α blockade on cytokines production in treated Tb group. Level of cytokines after granuloma formation, *in vitro*, Treated Tb group (A-F) under different culture conditions—Medium or Antigen Medium and Bead or BCG-Bead, in the presence or absence of Infliximab. *Statistical differences between the conditions tested after the addition of Infliximab, in the different cytokines. Wilcoxon and Kruskal-Wallis tests, p-value <0.05. The horizontal lines represent the medians, the bars represent the 25–75% percentiles and the vertical lines represent the 10–90% percentiles.

**Fig 5:**
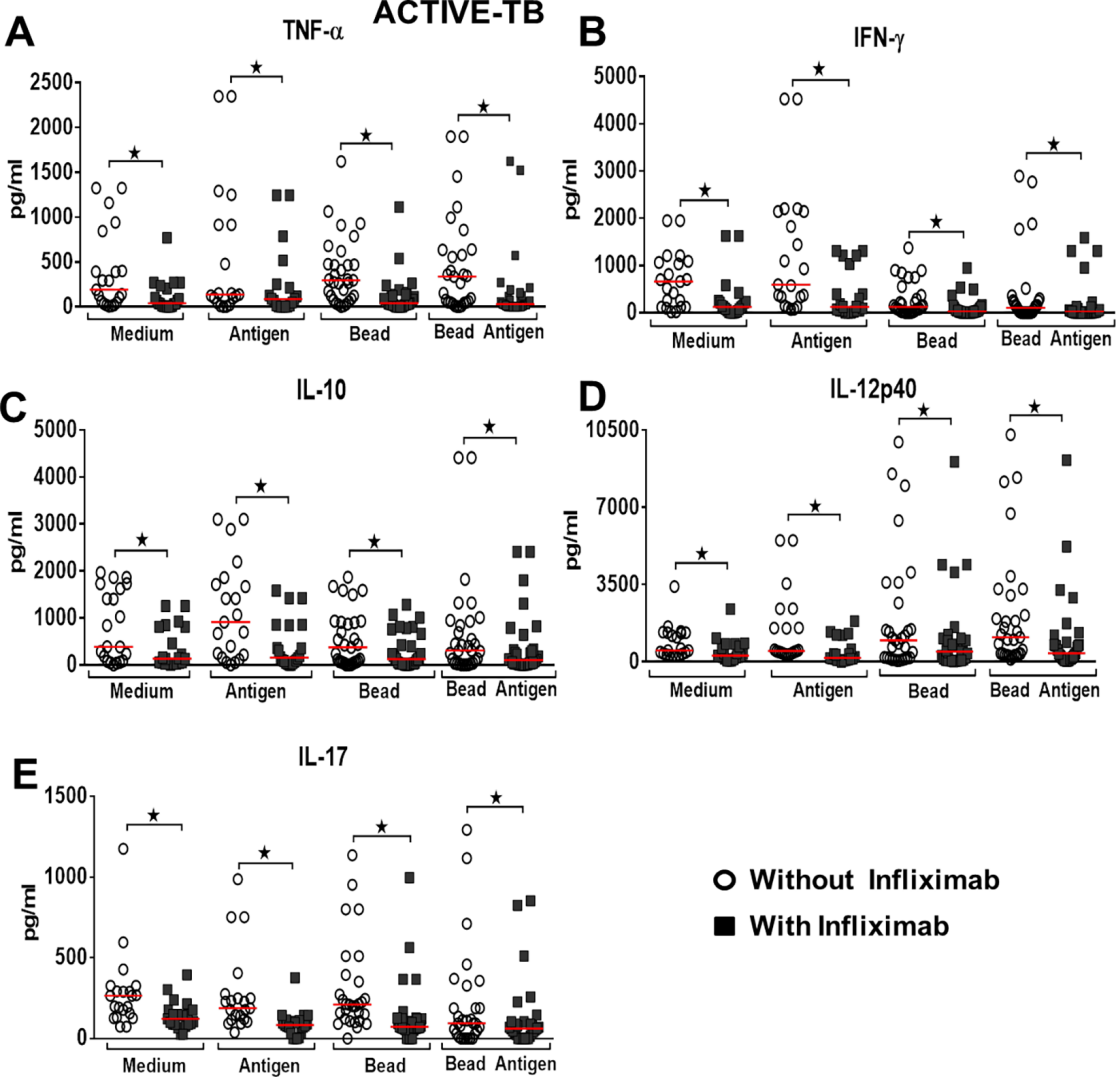
Effect of TNF-α blockade on cytokines production in active Tb group. Level of cytokines after granuloma formation, *in vitro*, Active Tb group (A-F) under different culture conditions—Medium or Antigen Medium and Bead or BCG-Bead, in the presence or absence of Infliximab. *Statistical differences between the conditions tested after the addition of Infliximab, in the different cytokines. Wilcoxon and Kruskal-Wallis tests, p-value <0.05. The horizontal lines represent the medians, the bars represent the 25–75% percentiles and the vertical lines represent the 10–90% percentiles.

After determining the capacity for granuloma formation, in vitro, we sought to evaluate the effect of TNF-α blockade in the levels of cytokines with potential role in promoting this reaction. Cytokines were evaluated on the 5^th^ day of culture. The TNF-α blockade, by using infliximab, was effective and significantly decreased cytokine levels in the four conditions tested and in all groups of patients studied (Figs 3–5). In individuals in the PPD+ control group, TNF-α blockade significantly reduced IFN-γ levels only in the BCG-bead condition (p = 0.034) (Wilcoxon), (Fig 3B). IL-10 levels, following TNF-α blockade, decreased significantly in cultures with Medium, Antigen and non-conjugated bead (p = 0.031, p = 0.015, p = 0.025) (Wilcoxon), (Fig 3C). IL-12p40 levels decreased significantly at all culture conditions after TNF-α blockade (p = 0.015, 0.015, 0.006, 0.001), (Wilcoxon), (Fig 3D). IL-17 levels significantly decreased in the Medium, bead and BCG-bead conditions after TNF-α blockade (p = 0.015, p = 0.001, p = 0.003), (Wilcoxon), (Fig 3E).

In patients in the treated TB group, TNF-α blockade significantly reduced IFN-γ levels in the four conditions tested (p = 0.001, p = 0.002, p = 0.001, p = 0.001), (Wilcoxon), (Fig 4B). IL-10 levels after TNF-α blockade were significantly decreased in the four conditions (p = 0.004, p = 0.002, p = 0.005, p = 0.001), (Wilcoxon), (Fig 4C). IL-12p40 levels decreased significantly at all culture conditions after TNF-α blockade (p = 0.004, p = 0.004, p = 0.001, p = 0.001), (Wilcoxon), (Fig 4D). IL-17 levels significantly decreased at all culture conditions after TNF-α blockade (p = 0.004, p = 0.005, p = 0.005, p = 0.001), (Wilcoxon), (Fig 4E).

In patients in the active TB group, TNF-α blockade significantly reduced IFN-γ levels in the four conditions tested (p = 0.001, p = 0.001, p = 0.001, p = 0.001), (Wilcoxon), (Fig 5B). IL-10 levels after TNF-α blockade significantly decreased in the four conditions (p = 0.001, p = 0.001, p = 0.001, p = 0.001), (Wilcoxon), (Fig 5C). IL-12p40 levels decreased significantly at all culture conditions after TNF-α blockade (p = 0.001, p = 0.001, p = 0.001, p = 0.001), (Wilcoxon), (Fig 5D). IL-17 levels significantly decreased at all culture conditions after TNF-α blockade (p = 0.001, p = 0.001, p = 0.001, p = 0.003), (Wilcoxon), (Fig 5E).

## Discussion

In the present study, in an *in vitro* model of granulomatous reaction, we evaluated the effects of TNF-α inhibition on the organization of granuloma and the production of prototypical response cytokines Th1, Th17 and regulatory T in three groups of patients. The *in vitro* model of granuloma allows us to study the initial steps in the formation and maintenance of granulomas, facilitating the approach of translational aspects of human *M*.*tuberculosis* infection. However, they do not have the pulmonary structure and the conditions of the complete tissue microenvironment, being one of the limitations of the study [23]. Our data point to a significant inhibitory effect of TNF-α blockade in the group of patients with treated tuberculosis, both in the formation of granuloma and in the production of the cytokines tested. TNF-α blockade in patients with active tuberculosis and in the PPD positive control group did not interfere with the formation of granuloma, but modulated some of the cytokines analyzed. The chronic character and maintenance of the latency state of tuberculosis depend on several immune mechanisms involved in this process, such as the activation of macrophages and the formation and maintenance of granulomas that have a key role in the containment of the infection [24–26]. Although situations of evident immunosuppression are directly linked to the reactivation of tuberculosis, it is not always possible to determine which immune changes lead to the reactivation of the disease. Several situations cause the individual to develop immunosuppression, and in recent years one of them is highlighted: the use of immunobiologicals drugs for the treatment of chronic inflammatory diseases, such as TNF-α blockers [11].

We have shown that patients clinically cured of tuberculosis have granuloma indices significantly higher than the others. In these patients, TNF-α blockade significantly reduced granuloma formation in vitro, making it with similar rates to the other groups. In experimental models with zebrafish, TNF-α deficiency leads to the formation of a disorganized granuloma with increased mycobacteria growth and macrophages necrosis [27]. In humans, therapeutic blockade of TNF-α leads to an increased risk of reactivation of some diseases, especially tuberculosis [28].

In this study, we also analyzed the effect of TNF-α blockade on the modulation of Th1, Th17, and regulatory T cytokines both in the granuloma model and after the stimulation with soluble antigens. We also evaluated IL-4 and LT-α, but with undetectable dosages in the culture supernatants, and IL-9 cytokine was not evaluated in this study. The dose of Infliximab used significantly reduced TNF-α levels in the supernatants at all culture conditions, indicating the efficacy of the concentration used to reduce the bioavailability of this cytokine. TNF-α is a proinflammatory cytokine, predominantly produced and secreted by monocytes, macrophages and T lymphocytes and its predominant effects on mycobacterial infection are the maintenance of granuloma structure, increase in adhesion molecule expression, production of reactive oxygen and nitrogen intermediates and increased expression of chemokines such as CCL-2, CCL5, CCL9 and CXCL10 [9,29–32].

Although in individuals PPD positive control, TNF-α blockade did not significantly alter the granuloma index, the blockade was shown to significantly reduce IL-12p40 levels in all conditions of antigenic stimulation, solubilized and immobilized in bead. IFN-γ and IL-17 were modulated negatively only in the condition of antigen immobilized in bead and IL-10 only when culture-soluble antigen was used. The granuloma index was not affected by TNF-α blockade in the active disease group, but significantly reduced the levels of IFN-γ, IL-12p40, IL-17 and IL-10 in all antigenic stimulus conditions tested. In the group of patients with treated tuberculosis, a reduction in all cytokines tested was verified. There was also a significant reduction in the granuloma index.

Interestingly, although cytokine levels were similar between the active disease and after treatment groups, the granuloma formation in the latter was significantly greater. The ability to form granuloma in the treated TB group was not exclusively associated with the cytokine pattern analyzed in this study. This suggests the involvement of other cytokines or chemokines, as well as adhesion molecules that may be directly responsible for intercellular adhesion in the formation of granuloma [33–35] or downstream pathways in the action of these and other cytokines, which may reflect different immune responses triggered in environments with similar concentrations of cytokines, especially TNF-α [36].

*In vitro* blockade of TNF-α with Infliximab led to a negative modulation of important cytokines in *M*. *tuberculosis* infection. Experimental studies in primate models and in human disease make clear the importance of the IL12/IFN-γ axis and the IL-10/TNF-α balance in the control of *M*. *tuberculosis* infection [37,38]. Carriers of innate errors in the IL-12/23-IFN-γ axis are more susceptible to *M*. *tuberculosis* infections and also by atypical mycobacteria [39]. IFN-γ and TNF-α are crucial for the complete activation of bactericidal functions of the macrophage producing NO and restricting the growth of mycobacteria [31,40–42].

The presence of IL-17 producing T cells during a secondary immune response in the lung environment has been shown to be important for protection against active tuberculosis [43]. Negative modulation of IL-17 promoted by the in vitro blockade of TNF-α may also be implicated in the risk of reactivation of *M tuberculosis* infection in treated patients [44]. Similarly, a negative modulation of IL-10 was observed, which despite being described as a regulatory cytokine has been associated with the clinical cure of patients with tuberculosis [38] and experimental models have associated its production with IFN-γ/IL-10—double producers T cells [45]. The introduction of immunobiologics in the therapy of autoimmune diseases such as: rheumatoid arthritis, juvenile idiopathic arthritis as well as in Inflammatory Bowel Diseases, has brought about an improvement in the therapeutic response and quality of life of these patients [11]. Nevertheless, modulation of the immune response may impair the fragile balance of the microorganism/host relationship in some latent or subclinical infectious diseases, increasing in these patients the risk of reactivation of latent tuberculosis [15].

In this study using in vitro granuloma model, we observed that TNF-α blockade not only blocks its bioavailability, with significant modulation of granuloma, but also significantly reduces the levels of IL-12p40, IFN-γ, IL-17 and IL-10. The participation of IL-10 in the formation of granuloma in tuberculosis requires further studies, since this cytokine is associated with the clinical cure of the disease and in the repertoire of antigen-specific T cells against *M*. *tuberculosis* [38]. The negative modulation of TNF-α, IFN-γ, IL-10, IL-12p40, IL-17, may be associated with the reactivation of the infection observed in patients taking Infliximab or other TNF-α blockers, which increases the importance of screening for latent tuberculosis in patients with chronic inflammatory diseases candidates for the use of immunobiological TNF blockers.

## Supporting information

S1 Table Demographic data. (PDF)

S2 Table Granuloma index. (PDF)

S3 Table Cytokine levels. (PDF)

## References

[1] Restrepo BI, Schlesinger LS (2014) Impact of diabetes on the natural history of tuberculosis. Diabetes Res Clin Pract. 10.1016/j.diabres.2014.06.011 PMC4260985 25082309

[2] Alsdurf H, Hill PC, Matteelli A, Getahun H, Menzies D (2016) The cascade of care in diagnosis and treatment of latent tuberculosis infection: a systematic review and meta-analysis. Lancet Infect Dis 16: 1269–1278. doi: 10.1016/S1473-3099(16)30216-X 27522233 10.1016/S1473-3099(16)30216-X

[3] Hartman-Adams H, Clark K, Juckett G (2014) Update on latent tuberculosis infection. Am Fam Physician 89: 889–896. 25077395

[4] Eurosurveillance editorial t (2013) WHO publishes Global tuberculosis report 2013. Euro Surveill 18. 24176622

[5] Caruso AM, Serbina N, Klein E, Triebold K, Bloom BR, et al (1999) Mice deficient in CD4 T cells have only transiently diminished levels of IFN-gamma, yet succumb to tuberculosis. J Immunol 162: 5407–5416. 10228018

[6] Mogues T, Goodrich ME, Ryan L, LaCourse R, North RJ (2001) The relative importance of T cell subsets in immunity and immunopathology of airborne Mycobacterium tuberculosis infection in mice. J Exp Med 193: 271–280. 11157048 10.1084/jem.193.3.271 PMC2195922

[7] O'Garra A, Redford PS, McNab FW, Bloom CI, Wilkinson RJ, et al (2013) The immune response in tuberculosis. Annu Rev Immunol 31: 475–527. doi: 10.1146/annurev-immunol-032712-095939 23516984 10.1146/annurev-immunol-032712-095939

[8] Cosma CL, Sherman DR, Ramakrishnan L (2003) The secret lives of the pathogenic mycobacteria. Annu Rev Microbiol 57: 641–676. doi: 10.1146/annurev.micro.57.030502.091033 14527294 10.1146/annurev.micro.57.030502.091033

[9] Flynn JL, Chan J (2005) What's good for the host is good for the bug. Trends Microbiol 13: 98–102. doi: 10.1016/j.tim.2005.01.005 15737727 10.1016/j.tim.2005.01.005

[10] Flynn JL, Chan J (2001) Tuberculosis: latency and reactivation. Infect Immun 69: 4195–4201. doi: 10.1128/IAI.69.7.4195-4201.2001 11401954 10.1128/IAI.69.7.4195-4201.2001 PMC98451

[11] Yasui K (2014) Immunity against Mycobacterium tuberculosis and the risk of biologic anti-TNF-alpha reagents. Pediatr Rheumatol Online J 12: 45 doi: 10.1186/1546-0096-12-45 25317081 10.1186/1546-0096-12-45 PMC4196001

[12] Kornbluth A (1998) Infliximab approved for use in Crohn's disease: a report on the FDA GI Advisory Committee conference. Inflamm Bowel Dis 4: 328–329. 9836088 10.1002/ibd.3780040415

[13] Schuna AA, Megeff C (2000) New drugs for the treatment of rheumatoid arthritis. Am J Health Syst Pharm 57: 225–234. 10674776 10.1093/ajhp/57.3.225

[14] Doran MF, Crowson CS, Pond GR, O'Fallon WM, Gabriel SE (2002) Frequency of infection in patients with rheumatoid arthritis compared with controls: a population-based study. Arthritis Rheum 46: 2287–2293. doi: 10.1002/art.10524 12355475 10.1002/art.10524

[15] Seong SS, Choi CB, Woo JH, Bae KW, Joung CL, et al (2007) Incidence of tuberculosis in Korean patients with rheumatoid arthritis (RA): effects of RA itself and of tumor necrosis factor blockers. J Rheumatol 34: 706–711. 17309133

[16] Martin-Mola E, Balsa A (2009) Infectious complications of biologic agents. Rheum Dis Clin North Am 35: 183–199. doi: 10.1016/j.rdc.2009.03.009 19481004 10.1016/j.rdc.2009.03.009

[17] Puissegur MP, Botanch C, Duteyrat JL, Delsol G, Caratero C, et al (2004) An in vitro dual model of mycobacterial granulomas to investigate the molecular interactions between mycobacteria and human host cells. Cell Microbiol 6: 423–433. doi: 10.1111/j.1462-5822.2004.00371.x 15056213 10.1111/j.1462-5822.2004.00371.x

[18] Crouser ED, White P, Guirado Caceres E, Julian MW, Papp AC, et al (2017) A Novel In Vitro Human Granuloma Model of Sarcoidosis and Latent TB Infection. Am J Respir Cell Mol Biol. 10.1165/rcmb.2016-0321OC PMC5650085 28598206

[19] Boehme CC, Nabeta P, Hillemann D, Nicol MP, Shenai S, et al (2010) Rapid molecular detection of tuberculosis and rifampin resistance. N Engl J Med 363: 1005–1015. doi: 10.1056/NEJMoa0907847 20825313 10.1056/NEJMoa0907847 PMC2947799

[20] Sotgiu G, Sulis G, Matteelli A (2017) Tuberculosis-a World Health Organization Perspective. Microbiol Spectr 5. 10.1128/microbiolspec.tnmi7-0036-2016 PMC11687456 28185618

[21] Silva-Teixeira DN, Ferreira MG, Nogueira-Machado JA, Doughty BL, Goes AM (1993) Human giant cell formation induced in vitro by Schistosoma mansoni antigens. Braz J Med Biol Res 26: 609–613. 8257946

[22] Doughty BL, Goes AM, Parra JC, Rocha RS, Katz N, et al (1987) Granulomatous hypersensitivity to Schistosoma mansoni egg antigens in human schistosomiasis. I. Granuloma formation and modulation around polyacrylamide antigen-conjugated beads. Mem Inst Oswaldo Cruz 82 Suppl 4: 47–54. 3151115 10.1590/s0074-02761987000800009

[23] Guirado E, Schlesinger LS (2013) Modeling the Mycobacterium tuberculosis Granuloma—the Critical Battlefield in Host Immunity and Disease. Front Immunol 4: 98 doi: 10.3389/fimmu.2013.00098 23626591 10.3389/fimmu.2013.00098 PMC3631743

[24] Parrish NM, Dick JD, Bishai WR (1998) Mechanisms of latency in Mycobacterium tuberculosis. Trends Microbiol 6: 107–112. 9582936 10.1016/s0966-842x(98)01216-5

[25] Saunders BM, Britton WJ (2007) Life and death in the granuloma: immunopathology of tuberculosis. Immunol Cell Biol 85: 103–111. doi: 10.1038/sj.icb.7100027 17213830 10.1038/sj.icb.7100027

[26] Schreiber HA, Sandor M (2010) The role of dendritic cells in mycobacterium-induced granulomas. Immunol Lett 130: 26–31. doi: 10.1016/j.imlet.2009.12.009 20005900 10.1016/j.imlet.2009.12.009 PMC3174523

[27] Clay H, Volkman HE, Ramakrishnan L (2008) Tumor necrosis factor signaling mediates resistance to mycobacteria by inhibiting bacterial growth and macrophage death. Immunity 29: 283–294. doi: 10.1016/j.immuni.2008.06.011 18691913 10.1016/j.immuni.2008.06.011 PMC3136176

[28] Minozzi S, Bonovas S, Lytras T, Pecoraro V, Gonzalez-Lorenzo M, et al (2016) Risk of infections using anti-TNF agents in rheumatoid arthritis, psoriatic arthritis, and ankylosing spondylitis: a systematic review and meta-analysis. Expert Opin Drug Saf 15: 11–34. doi: 10.1080/14740338.2016.1240783 27924643 10.1080/14740338.2016.1240783

[29] Nathan C, Shiloh MU (2000) Reactive oxygen and nitrogen intermediates in the relationship between mammalian hosts and microbial pathogens. Proc Natl Acad Sci U S A 97: 8841–8848. 10922044 10.1073/pnas.97.16.8841 PMC34021

[30] Zahrt TC, Deretic V (2002) Reactive nitrogen and oxygen intermediates and bacterial defenses: unusual adaptations in Mycobacterium tuberculosis. Antioxid Redox Signal 4: 141–159. doi: 10.1089/152308602753625924 11970850 10.1089/152308602753625924

[31] Flynn JL, Goldstein MM, Triebold KJ, Sypek J, Wolf S, et al (1995) IL-12 increases resistance of BALB/c mice to Mycobacterium tuberculosis infection. J Immunol 155: 2515–2524. 7650381

[32] Bean AG, Roach DR, Briscoe H, France MP, Korner H, et al (1999) Structural deficiencies in granuloma formation in TNF gene-targeted mice underlie the heightened susceptibility to aerosol Mycobacterium tuberculosis infection, which is not compensated for by lymphotoxin. J Immunol 162: 3504–3511. 10092807

[33] Algood HM, Chan J, Flynn JL (2003) Chemokines and tuberculosis. Cytokine Growth Factor Rev 14: 467–477. 14563349 10.1016/s1359-6101(03)00054-6

[34] Mendez-Samperio P (2008) Expression and regulation of chemokines in mycobacterial infection. J Infect 57: 374–384. doi: 10.1016/j.jinf.2008.08.010 18838171 10.1016/j.jinf.2008.08.010

[35] Newton SM, Mackie SL, Martineau AR, Wilkinson KA, Kampmann B, et al (2008) Reduction of chemokine secretion in response to mycobacteria in infliximab-treated patients. Clin Vaccine Immunol 15: 506–512. doi: 10.1128/CVI.00401-07 18160618 10.1128/CVI.00401-07 PMC2268261

[36] McFarlane SM, Pashmi G, Connell MC, Littlejohn AF, Tucker SJ, et al (2002) Differential activation of nuclear factor-kappaB by tumour necrosis factor receptor subtypes. TNFR1 predominates whereas TNFR2 activates transcription poorly. FEBS Lett 515: 119–126. 11943206 10.1016/s0014-5793(02)02450-x

[37] Cepeda M, Salas M, Folwarczny J, Leandro AC, Hodara VL, et al (2013) Establishment of a neonatal rhesus macaque model to study Mycobacterium tuberculosis infection. Tuberculosis (Edinb) 93 Suppl: S51–59. 24388650 10.1016/S1472-9792(13)70011-8 PMC4051704

[38] da Silva MV, Figueiredo AA, Machado JR, Castellano LC, Alexandre PB, et al (2013) T Cell Activation and Proinflammatory Cytokine Production in Clinically Cured Tuberculosis Are Time-Dependent and Accompanied by Upregulation of IL-10. PLoS One 8: e65492 doi: 10.1371/journal.pone.0065492 23824716 10.1371/journal.pone.0065492 PMC3688829

[39] Pedraza-Sanchez S, Herrera-Barrios MT, Aldana-Vergara R, Neumann-Ordonez M, Gonzalez-Hernandez Y, et al (2010) Bacille Calmette-Guerin infection and disease with fatal outcome associated with a point mutation in the interleukin-12/interleukin-23 receptor beta-1 chain in two Mexican families. Int J Infect Dis 14 Suppl 3: e256–260. 20171917 10.1016/j.ijid.2009.11.005

[40] MacMicking JD, North RJ, LaCourse R, Mudgett JS, Shah SK, et al (1997) Identification of nitric oxide synthase as a protective locus against tuberculosis. Proc Natl Acad Sci U S A 94: 5243–5248. 9144222 10.1073/pnas.94.10.5243 PMC24663

[41] MacMicking JD, Taylor GA, McKinney JD (2003) Immune control of tuberculosis by IFN-gamma-inducible LRG-47. Science 302: 654–659. doi: 10.1126/science.1088063 14576437 10.1126/science.1088063

[42] Flynn JL, Chan J, Triebold KJ, Dalton DK, Stewart TA, et al (1993) An essential role for interferon gamma in resistance to Mycobacterium tuberculosis infection. J Exp Med 178: 2249–2254. 7504064 10.1084/jem.178.6.2249 PMC2191274

[43] Dheda K, Chang JS, Lala S, Huggett JF, Zumla A, et al (2008) Gene expression of IL17 and IL23 in the lungs of patients with active tuberculosis. Thorax 63: 566–568. 10.1136/thx.2007.092205 18511642

[44] da Silva MV, Massaro Junior VJ, Machado JR, Silva DAA, Castellano L, et al (2015) Expression Pattern of Transcription Factors and Intracellular Cytokines Reveals That Clinically Cured Tuberculosis Is Accompanied by an Increase in Mycobacterium-Specific Th1, Th2, and Th17 Cells. BioMed Research International. 10.1155/2015/591237 PMC4427018 26000298

[45] Jankovic D, Kullberg MC, Feng CG, Goldszmid RS, Collazo CM, et al (2007) Conventional T-bet(+)Foxp3(-) Th1 cells are the major source of host-protective regulatory IL-10 during intracellular protozoan infection. J Exp Med 204: 273–283. doi: 10.1084/jem.20062175 17283209 10.1084/jem.20062175 PMC2118735

