# Supplementary material for: TNF-α blockade impairs in vitro tuberculous granuloma formation and down modulate Th1, Th17 and Treg cytokines
Source: PLoS One. 2018 Mar 15;13(3):e0194430. doi: 10.1371/journal.pone.0194430 (PMC5854376; doi:10.1371/journal.pone.0194430)
Supplement: S1 Table — (PDF) [file pone.0194430.s001.pdf]

Gender	Clinical form	Age
Male	Active	65
Male	Active	52
Male	Active	18
Male	Active	64
Male	Active	33
Male	Active	34
Male	Active	34
Male	Active	36
Male	Active	70
Male	Active	56
Male	Active	44
Male	Active	54
Male	Active	73
Male	Active	27
Male	Active	64
Male	Active	39
Male	Active	62
Male	Active	36
Male	Active	26
Female	Active	65
Female	Active	34
Female	Active	27
Female	Active	59
Female	Active	66
Female	Active	77
Female	Active	30
Female	Active	79
Female	Active	60
Female	Active	69
Female	Active	25
Female	Active	25
Male	Treated	85
Male	Treated	60
Male	Treated	79
Male	Treated	62
Male	Treated	49
Male	Treated	47
Male	Treated	37
Male	Treated	28
Male	Treated	46
Female	Treated	32
Female	Treated	40
Female	Treated	80
Female	Treated	30
Female	Treated	54
Female	Treated	50
Female	Treated	70
Female	Treated	29
Female	Treated	27

Female	Treated	26
Female	Treated	43
Female	Treated	47
Female	Treated	23
Male	PPD	19
Male	PPD	46
Male	PPD	33
Male	PPD	63
Female	PPD	21
Female	PPD	34
Female	PPD	30
Female	PPD	51
Male	Treated	19
Female	Treated	46
Female	Treated	33
Female	Treated	63
Female	Treated	21
