# Supplementary material for: TNF-α blockade impairs in vitro tuberculous granuloma formation and down modulate Th1, Th17 and Treg cytokines
Source: PLoS One. 2018 Mar 15;13(3):e0194430. doi: 10.1371/journal.pone.0194430 (PMC5854376; doi:10.1371/journal.pone.0194430)
Supplement: S2 Table — (PDF) [file pone.0194430.s002.pdf]

Granuloma index Bead Antigen 5 days

BEAD ANTIGEN PPD	BEAD ANTIGEN PPD INFLIX	BEAD ANTIGEN ACTIVE DISEASE	BEAD ANTIGEN ACTIVED DISEASE INFLIX	BEAD ANTIGEN TREATED
3.06	2.88	2.4	2.3	2.68
2.92	2.16	2.46	2.68	2.18
3.22	3	2.2	2.1	3.74
2.42	2.36	2.32	2.32	3.6
2.2	2.32	2.58	2.96	3.76
2.24	2.14	2.1	2.32	3.48
2.24	2.74	2.9	3.22	3.18
2.16	2.3	2.4	2.32	3.66
2.58	2.18	2.8	2.9	3.5

Granuloma index Bead Antigen 10 days

BEAD ANTIGEN PPD	BEAD ANTIGEN PPD INFLIX	BEAD ANTIGEN ACTIVE DISEASE	BEAD ANTIGEN ACTIVE DISEASE INFLIX	BEAD ANTIGEN TREATED
2.54	2.92	2.98	2.28	3
2.8	2.86	2.94	2.32	2.26
2.92	2.8	3	2.54	3.6
2.82	3.06	2.94	3	2.64
2.96	3.26	2.36	2.18	3.08
3.46	3.56	2.18	2.42	3.54
2.88	2.94	3.68	4.1	2.94
3	2.58	2.68	2.84	3
2.95	2.77	2.3	2.34	2.7

BEAD ANTIGEN TREATED INFLIX
2.68
2.38
3.1
2.9
2.78
2.88
2.72
2.22
2.39

BEAD ANTIGEN TREATED INFLIX
3.12
2.48
3.24
2.62
2.62
2.42
2.2
2.34
2.13
