# Supplementary material for: TNF-α blockade impairs in vitro tuberculous granuloma formation and down modulate Th1, Th17 and Treg cytokines
Source: PLoS One. 2018 Mar 15;13(3):e0194430. doi: 10.1371/journal.pone.0194430 (PMC5854376; doi:10.1371/journal.pone.0194430)
Supplement: S3 Table — (PDF) [file pone.0194430.s003.pdf]

PPD TNF

MEDIUM	MEDIUM INFLIX	ANTIGEN	ANTIGEN INFLIX	BEAD
				386.404
				421.329
				16.45
				12.366
				19.14
				23.614
				191.967
440.847	129.126	480.249	41.898	
				336.303
		364.134	34.134	110.262
175.611	38.901	186.072	22.107	95.478
391.512	44.073	334.095	47.229	122.841
70.095	0	37.287	0.291	518.088
50.787	0.291	31.98	11.979	96.831
155.334	8.667	166.92	35.271	386.404

Treated TNF

MEDIUM	MEDIUM INFLIX	ANTIGEN	ANTIGEN INFLIX	BEAD
				168.822
				288.834
				147.246
				67.671
				106.128
				125.565
				82.026
				80.511
				273.805
				463.608
113.778	99.588	420.264	128.331	382.009
142.701	43.047	0	0	
379.338	14.481	29.508	0	
181.188	115.449	68.64	71.565	342.868
126.135	68.64			
192.183	71.565	99.15	75.51	719.406
127.848	67.671	68.64	65.265	62.477
87.126	61.923			39.599
24.033	0	39.068	27.249	44.022
58.271	28.177	65.015	57.228	79.889

16.415	0	36.959	28.642	47.002
70.775	22.934	74.658	3.687	568.822
48.218	14.538	105.593	0	144.022
213.778	199.588	320.264	228.331	239.599
46.415	2.36	66.959	28.642	653.312
58.271	28.177	145.015	57.228	247.002
148.218	84.538	905.593	139.36	273.805

Active disease TNF

MEDIUM	MEDIUM INFLIX	ANTIGEN	ANTIGEN INFLIX	BEAD
				173.508
				620.202
				59.217
				304.107
				37.392
				87.564
				407.052
				188.217
				294.954
				1.062.617
291.078	269.007			265.602
1.323.861	68.118	2.348.133	1.242.699	928.983
34.134	29.835	99.87	68.049	463.511
140.649	68.049	149.067	115.26	74.064
63.054	38.484	135.108	123.15	468.441
45.108	33.054	92.304	84.888	679.681
390.111	203.421	118.482	89.772	356.476
189.738	11.475	0	0	24.226
399.348	101.853	20.49	0	122.333
20.414	6.122	44.022	15.098	260.568
9.068	7.8	914.239	11.201	910.12
292.191	1.035	475.918	250.876	26.875
1159.93	33.298	122.553	45.185	0.123
102.354	0	2.829	0	10.078
19.164	0	8.245	0	273.508
6.075	0	148.085	0	122.333
391.078	269.007			304.107
129.068	87.8	914.239	211.201	1.620.202
940.649	268.049	1.249.067	515.26	787.564
1.323.861	768.118	2.348.133	1.242.699	474.064
845.108	233.054	1.292.304	784.888	463.511

PPD IFN

MEDIUM	MEDIUM INFLIX	ANTIGEN	ANTIGEN INFLIX	BEAD
				230.163
				132.253
				202.973
				25.36
				47.956
				12.367
				0
0	0	0	0	280.508
525.65	241.156	867.937	85.415	164.559
		389.901	123.402	259.326
20.844	49.716	38.091	23.703	569.61
0	0	79.215	76.236	165.147
330.816	88.188	0	604.761	293.025
203.478	67.341	61.44	91.191	55.566
127.719	935.088	474.633	70.299	230.163

Treated IFN

MEDIUM	MEDIUM INFLIX	ANTIGEN	ANTIGEN INFLIX	BEAD
				433.373
				664.926
				546.471
				152.412
				110.403
				142.503
				88.269
				68.655
				20.871
				217.089
431.879	323.909	1.628.866	227.81	31.511
1.551.633	311.706	120.789	69.708	0
691.143	0	105.705	63.096	10.091
218.421	141.267			0
101.784	62.538			21.58
78.453	66.207	952.026	122.73	261.098
82.131	60.093	182.223	46.659	27.155
52.761	64.983			24.308
34.777	18.555	30.971	83.868	22.948
28.378	16.961	125.803	19.947	53.188
37.982	17.513	56.379	17.585	424.248

65.738	29.076	468.307	460.646	333.373
683.706	30.368	845.979	42.318	22.948
531.879	323.909	1.328.866	527.81	24.308
37.982	17.513	56.379	17.585	321.58
28.378	16.961	125.803	19.947	424.248
683.706	130.368	845.979	242.318	90.871

Active disease IFN

MEDIUM	MEDIUM INFLIX	ANTIGEN	ANTIGEN INFLIX	BEAD
				0
				0
				0
				66.729
				553.428
				843.33
				899.523
				747.822
				67.548
118.285	0	369.078	0	103.211
1.950.702	1.626.195	2.204.711	1209.8	20.055
97.898	0	143.686	0	7.116
258.627	211.638	4531.53	1.309.257	149.899
1.090.575	54.861	1.828.992	1.030.419	79.999
1.207.134	143.052	2.145.084	389.901	289.175
1032.81	581.649	572.859	293.868	85.804
660.552	253.668	594.822	51.39	17.656
1067.04	119.055	69.071	0	18.097
22.446	15.831	76.187	36.125	33.42
18.712	15.545	334.29	19.947	33.577
704.95	112.468	105.661	41.906	212.369
418.788	28	122.259	47.121	186.152
701.69	229.744	929.892	69.094	747.612
513.713	72.261	1.097.087	94.546	136.369
812.372	421.362	1.443.055	176.28	218.097
118.285	0	369.078	125.326	366.729
118.712	85.545	334.29	119.947	893.369
258.627	211.638	4531.53	1.309.257	1375.23
1.950.702	1.626.195	2.204.711	1209.8	149.899
1.207.134	143.052	2.145.084	389.901	77.116

PPD IL-10

MEDIUM	MEDIUM INFLIX	ANTIGEN	ANTIGEN INFLIX	BEAD
				240.291
				2.217.588
				1.065.885
				62.991
				25.364
				67.514
				22.747
				1.435.296
				546.132
1.001.178	621.063	1.864.272	1.493.211	113.484
		3.480.225	871.077	339.546
63.21	22.272	330.156	0	695.358
300.303	81.336	689.529	133.194	97.272
105.984	75.897	360.078	30.15	635.466
59.814	9.249	453.327	0	446.337
718.449	371.949	290.412	63.663	640.291

Treated IL-10

MEDIUM	MEDIUM INFLIX	ANTIGEN	ANTIGEN INFLIX	BEAD
				3.569.826
				55.686
				57.621
				91.84
				23.369
				91.84
				0
				1.957
				35.321
				433.053
2.636.364	2403.93	2.533.029	1.516.527	772.797
36.489	18.453	46.566	18.453	326.325
67.314	19.398	32.676	9.981	62.703
94.588	128.934			5.698
0	0			250.127
322.646	131.682	236.094	215.486	617.105
28.06	0	51.415	19.13	54.31
28.06	0			48.616
69.554	37.11	61.942	167.736	45.896
56.756	33.922	251.606	39.894	106.376
75.964	35.026	112.758	35.17	848.496

131.476	58.152	936.614	921.292	2.569.826
100.253	46.712	1.691.958	84.636	925.896
2.636.364	2403.93	1.533.029	816.527	148.616
175.964	135.026	512.758	235.17	450.127
156.756	58.922	351.606	99.894	848.496
100.253	46.712	1.691.958	384.636	135.321

Active IL-10

MEDIUM	MEDIUM INFLIX	ANTIGEN	ANTIGEN INFLIX	BEAD
				1.586.994
				866.175
				1672.44
				0
				0
				0
				50.868
				53.757
				74.13
				898.802
1.730.244	1.253.571	1412.28	854.388	109.71
1.864.272	812.994	3.101.256	1.423.776	697.978
1.966.218	923.265	1.714.155	1.579.224	7.116
284.151	32.214	398.043	157.443	934.464
182.964	50.763	1.067.538	0	908.857
0	0	2.442	0	124.15
86.865	55.686	112.614	100.686	434.387
78.039	26.973	49.398	25.074	35.312
60.522	44.142	53.757	46.059	36.194
44.892	31.662	152.374	72.25	66.84
37.424	31.09	668.58	39.894	67.154
1409.9	224.936	211.322	83.812	424.738
837.576	56	244.518	94.242	372.304
1403.38	459.488	1.859.784	138.188	1.495.224
1.027.426	144.522	2.194.174	189.092	1.586.994
1.624.744	842.724	2886.11	352.56	536.194
1.730.244	1.253.571	1412.28	854.388	236.359
237.424	131.09	1668.58	839.894	1.866.175
384.151	132.214	698.043	357.443	550.868
1.864.272	812.994	3.101.256	1.423.776	934.464
386.865	155.686	912.614	300.686	87.116

PPD IL-12

MEDIUM	MEDIUM INFLIX	ANTIGEN	ANTIGEN INFLIX	BEAD
				4488.67
				2.415.572
				651.382
				399.422
				1101.52
				530.168
				443.594
				243.732
3.752.556	2730.36	1613.28	367.71	105.522
730.098	401.853	616.851	253.659	57.072
		386.586	176.586	174.948
272.694	237.784	447.228	291.274	562.988
824.526	517.158	858.61	733.066	0
31.848	11.556	39.148	39.148	8.322
524.6	273.498	100.828	59.32	362.988
624.526	217.158	758.61	533.066	4488.67

Treated IL-12

MEDIUM	MEDIUM INFLIX	ANTIGEN	ANTIGEN INFLIX	BEAD
				969.03
				142.626
				122.706
				91.84
				12.399
				91.84
				0
				3184.44
				6.289.432
711.381	442.65	429.177	257.652	626.658
315.468	175.278	300.522	111.939	2.580.028
447.132	333.003	174.378	78.912	2.201.786
94.588	128.934			3.935.188
0	0			2.691.226
322.646	131.682	236.094	215.486	
56.12	0	102.83	38.26	657.003
56.12	0			1461.35
411.249	16.273	978.037	115.501	706.824
1.102.932	634.316	414.683	428.471	4.183.458
498.703	545.51	611.824	13.534	2.497.922

3.212.822	1.719.089	1.852.769	527.589	969.03
1.945.764	1.370.357	3.032.644	2.079.445	1.706.824
711.381	442.65	429.177	257.652	1461.35
498.703	545.51	611.824	13.534	2.691.226
1.102.932	634.316	434.683	428.471	2.497.922
1.945.764	1.370.357	3.032.644	2.079.445	3184.44

Active disease IL-12

MEDIUM	MEDIUM INFLIX	ANTIGEN	ANTIGEN INFLIX	BEAD
				435.735
				190.314
				86.826
				130.272
				57.315
				91.977
				173.982
				184.203
				156.993
				9.964.572
594.105	212.454	533.001	149.781	4.052.404
316.851	135.735	541.755	223.335	8519.81
296.586	55.632	454.2	94.485	3.592.524
383.097	214.485	405.018	76.368	669.97
356.343	237.561	389.073	78.435	1.466.742
269.073	56.343	490.476	128.619	7.989.164
509.115	278.547	457.083	142.545	6407.09
441.879	376.44	430.752	166.872	205.81
515.667	356.55	352.548	81.939	63.644
421.566	268.711	350.369	0	667.082
297.72	196.56	5.505.263	353.706	1085.56
1114.62	120.735	324.162	45.177	1.394.346
1.178.555	620.584	1.538.911	1.201.803	1.066.241
1.349.908	1.069.185	1.520.533	571.616	2.672.936
1.324.719	391.256	324.192	317.035	1.435.735
3.415.103	2.390.009	2.413.128	1.835.662	1.363.644
1.594.105	812.454	1.533.001	349.781	830.272
1297.72	696.56	5.505.263	1.353.706	1.190.314
1.383.097	814.485	2.405.018	1.276.368	91.977
1.316.851	835.735	3.541.755	1.223.335	969.97
269.073	56.343	490.476	128.619	3.592.524

PPD IL- 17

MEDIUM	MEDIUM INFLIX	ANTIGEN	ANTIGEN INFLIX	BEAD
				207.77
				257.65
				138.354
				238.135
				10.258
				28.189
				9.258
				235.731
704.295	109.89	542.163	129.135	43.998
171.231	36.96	122.055	54.393	80.076
		0	0	125.072
148.13	73.654	45.896	0	123.738
164.842	0	0	0	61.424
95.02	0	0	0	95.02
20.578	0	0	0	29.644
44.72	0	0	0	207.77

Treated IL-17

MEDIUM	MEDIUM INFLIX	ANTIGEN	ANTIGEN INFLIX	BEAD
				165.231
				215.751
				282.018
				3.844
				21.532
				18.294
				19.912
				30.494
				0
				382.5
227.073	70.737	1.203.237	1.075.218	773.934
185.046	76.071	104.046	55.794	0
269.955	89.109	164.514	48.261	7.554
3.048	1.456			0
92.788	75.606			5.144
7.038	2.252	90.194	0	877.568
14.258	6.238	0	0	251.024
11.042	0			375.064
725.56	346.176	184.616	165.84	168.144
539.992	232.928	269.616	99.712	343.648
276.4	239.52	126.248	73.88	120.608

394.376	75.632	63.472	73.184	165.231
46.712	100.376	123.16	58.656	168.144
227.073	70.737	1.203.237	975.218	375.064
276.4	239.52	126.248	73.88	85.144
539.992	232.928	269.616	99.712	120.608
346.712	100.376	123.16	58.656	23.23

ctive disease IL-17

MEDIUM	MEDIUM INFLIX	ANTIGEN	ANTIGEN INFLIX	BEAD
				213.624
				353.334
				394.776
				800.904
				272.649
				201.096
				160.218
				225.279
				211.041
				69.96
326.565	177.564	249.135	143.823	162.87
196.149	103.677	187.563	76.389	0
292.107	122.103	103.971	83.19	510.859
265.275	149.643	92.697	32.046	
191.871	91.587	38.268	4.407	
73.971	26.532	228.486	0	100.722
427.809	238.209	177.621	0	109.548
198.663	65.769	177.954	95.199	246.144
290.757	92.751	232.785	107.679	91.024
156.576	141.304	141.304	91.024	151.648
126.248	88.144	752.992	71.056	107.92
290.168	100.376	146.416	80.528	239.304
130.856	75.632	120.608	110.448	178.56
1175.96	394.44	405.448	145.048	122.784
269.224	215.952	114.616	98.56	213.624
178.56	147.88	276.472	114.616	91.024
326.565	177.564	249.135	143.823	800.904
126.248	88.144	752.992	71.056	953.334
265.275	149.643	92.697	32.046	201.096
596.149	303.677	987.563	376.389	1.136.194
73.971	26.532	228.486	0	510.859

BEAD INFLIX	BEAD ANTIGEN	BCG ANTIGEN INFLIX
0	272.625	0
	142.164	0
0	53.924	0
0	31.599	28.323
	520.062	15.227
0	0	0
15.631	45.988	0
0	38.932	0
50.496		
	391.632	64.992
54.537	173.487	20.358
17.937	242.841	33.492
37.05	96.108	56.733
0	63.09	2.841
65.928	36.108	24.249
30.93	247.743	44.418
0	272.625	

BEAD INFLIX	BEAD ANTIGEN	BCG ANTIGEN INFLIX
75.252	380.187	45.246
12.978	37.029	0
37.029	0	0
61.923		
71.565		
76.008		
62.4	58.155	57.687
59.562		
0	165.947	0
76.65	468.441	0
50.675	290.427	0
		0
48.397	687.111	78.435
	0	0
25.684	710.423	17.513
0	3.222	0
0	60.366	54.639
24.49	163.362	9.918
45.185	89.867	75.065

33.298	34.714	33.883
275.252	480.187	145.246
54.49	363.362	19.918
128.36	360.366	154.639
348.075	758.369	256.39
93.298	432.714	233.883
123.6	165.947	25.369

**BEAD INFLIX**	**BEAD ANTIGEN**	**BCG ANTIGEN INFLIX**
54.42	1.898.079	1.622.358
112.305		
52.989	335.538	113.958
133.854	76.416	70.416
29.835	60.906	49.593
59.871	93.552	70.416
77.7	49.068	0
0	0	0
15.981	41.541	0
27.743	1.111.251	24.73
187.518	265.022	187.518
30.711	553.588	25.57
37.396	357.524	54.689
20.953	34.173	1.593
0	344.697	0
29.096	857.513	26.471
29.983	152.87	30.085
18.183	48.018	9.068
0	9.068	0
20.094	639.263	12.886
537.806	1453.94	63.115
0	9.332	0
0	0.02	0
0	48.09	0
154.42	1.898.079	1.522.358
89.369	399.068	123.589
133.854	576.416	270.416
1.112.305		
259.871	993.552	570.416
120.953	634.173	211.593
237.396	357.524	154.689

BEAD INFLIX	BEAD ANTIGEN	BEAD ANTIGEN INFLIX
0	521.908	0
0	734.291	0
0	675.926	34.992
	344.637	51.958
0	0	0
121.82	40.685	0
0	0	0
0	191.94	0
107.518	297.716	85.415
63.753	355.956	23.457
709.158	32.319	26.568
856.383	61.44	130.803
88.188	136.989	88.188
140.094	43.893	592.986
46.8	38.091	46.8
0	521.908	0

BEAD INFLIX	BEAD ANTIGEN	BCG ANTIGEN INFLIX
135.564	219.937	0
222.342	137.058	0
0	0	0
82.131		
64.983		
67.431		
69.879	42.999	46.659
58.869		
0	0	0
107.261	402.083	83.309
0	148.544	0
0	0	0
13.401		
0	0	0
186.516	53.516	0
27.616	287.776	21.19
17.656	28.254	24.094
16.762	31.376	19.193
16.961	24.964	16.894
42.943	358.679	22.163
31.054	89.429	37.287

235.564	219.937	98.326
16.961	24.964	16.894
16.762	31.376	19.193
186.516	553.516	125.36
131.054	689.429	237.287
23.6	123.6	45.369

**BEAD INFLIX**	**BEAD ANTIGEN**	**BCG ANTIGEN INFLIX**
0	0	0
0	0	0
0	0	0
45.621	1.772.271	1.309.257
106.281		
	83.178	50.859
360.675	123.555	82.995
159.537	10.677	0
0	0	0
20.152	295.193	0
0	0	0
0	0	0
0	0	0
40.44	32.446	0
22.164	16.517	0
0	513.166	0
0	0	0
15.715	18.712	15.658
15.949	18.791	15.658
28.116	65.136	51.176
21.638	149.961	38.386
61.356	220.647	16.277
47.976	143.667	22.53
534.708	152.024	59.421
89.369	256.369	123.369
115.949	358.791	215.658
145.621	2.772.271	1.309.257
493.369	2.893.369	1.589.363
944.918	1.883.178	950.859
40.44	132.446	23.693
23.369	248.369	112.699

BEAD INFLIX	BEAD ANTIGEN	BCG ANTIGEN INFLIX
608.544	223.163	652.218
213.491	2.507.358	289.898
2.794.734	850.198	2.297.778
30.941	210.09	182.265
	674.988	699.576
0	0	0
28.789	47.814	183.522
16.479	8.112	0
812.994	1.355.115	2.255.964
436.884	455.907	465.45
39.639	87.723	50.763
41.052	37.509	30.06
43.002	153.108	27.108
30.6	69.759	36.09
36.069	369.78	3.09
60.522	266.121	36.09
308.544	623.163	252.218

BEAD INFLIX	BEAD ANTIGEN	BCG ANTIGEN INFLIX
436.884		
7.167	17.292	9.981
7.167	3.423	0
56.12		
24.522		
78.102		
4.705	9.513	0
0		
28.269	33.171	55.484
271.023	365.15	246.512
540.275	934.91	711.063
522.633	369.422	159.161
75.696	61.221	0
9.337	5.86	13.961
308.945	80.05	0
472.106	702.44	417.856
35.312	56.508	48.188
33.524	62.752	38.386
33.922	49.928	33.788
85.886	717.358	44.326
62.108	178.858	74.574

436.884		
323.922	1.249.928	523.788
53.524	262.752	68.386
308.945	580.05	189
262.108	178.858	74.574
28.269	133.171	55.484

**BEAD INFLIX**	**BEAD ANTIGEN**	**BCG ANTIGEN INFLIX**
812.994	1.321.074	1.803.027
780.792	4.413.128	2.410.399
1.007.073	1.320.933	788.598
0	0	0
0	0	0
0	0	0
31.722	27.921	16.566
16.566	5.292	6.231
24.129	26.022	11.859
759.933	1010.44	640.642
154.602	92.736	179.31
421.403	396.542	244.887
5.666	22.827	14.017
192.533	243.514	104.148
660.988	950.269	822.474
31.754	419.061	33.646
157.676	436.171	313.116
31.43	37.424	31.316
31.898	37.582	31.316
56.232	130.272	102.352
43.276	299.922	76.772
122.712	441.294	32.554
95.952	287.334	45.06
1.069.416	304.048	118.842
812.994	1.821.074	1.303.027
231.898	837.582	331.316
125.369	689.36	258.369
1.280.792	4.413.128	2.410.399
231.722	927.921	216.566
392.533	543.514	294.148
25.666	122.827	54.017

BEAD INFLIX	BEAD ANTIGEN	BCG ANTIGEN INFLIX
1.249.246	3.219.046	0
1.488.906	2371.25	1329.39
0	559.624	0
137.3	441.568	163.816
	2.822.266	835.494
1.236.966	901.222	905.972
317.344	665.562	232.676
447.648	694.328	523.886
135.735	1.129.956	682.86
69.03	432.435	284.142
54.471	414.243	263.568
122.88	143.154	123.598
318.332	600.204	482.328
7.678	0	0
18.71	12.204	28.546
118.332	200.204	182.328
1.249.246	3.219.046	985.3

BEAD INFLIX	BEAD ANTIGEN	BCG ANTIGEN INFLIX
600.249	1.427.613	781.623
71.733	359.16	270.522
34.248	254.709	208.056
56.12		
24.522		
78.102		
9.41	19.026	0
1.801.428	6.475.802	1.670.322
3.048.956	2.258.498	2.657.434
503.172	1358.72	710.472
1493.54	3.924.818	1171.06
273.646	1.149.536	391.478
2.271.714	5.215.826	1.843.488
1.255.206		
152.358	2.148.136	115.501
262.324	1147.05	215.107
262.324	377.211	297.72
1.713.276	2.137.566	1.050.686
2.265.435	1695.84	1.358.733

600.249	1.427.613	781.623
562.324	877.211	297.72
262.324	1147.05	215.107
1.255.206	3.703.686	1.236.023
2.265.435	1695.84	1.358.733
1.801.428	6.475.802	1.670.322

**BEAD INFLIX**	**BEAD ANTIGEN**	**BCG ANTIGEN INFLIX**
269.697	824.097	306.468
144.825	1009.17	736.851
60.9	376.029	139.908
81.276	365.058	65.058
55.632	295.866	60.108
62.523	370.791	245.058
92.862	297.201	171.366
62.442	281.154	58.056
35.496	294.003	41.133
9.066.564	10294.63	9.135.216
1.573.724	3883.24	1.318.668
4.406.594	8161.98	5.224.554
1.064.764	3.301.188	726.506
775.46	538.568	383.56
425.426	1.002.408	323.108
4.399.774	8.356.878	3.253.738
4.052.404	6.718.136	2.907.662
184.304	199.638	130.215
10.802	114.519	72.166
48.236	1114.62	125.36
922.818	1.951.576	539.213
589.334	1.237.724	653.54
462.846	324.035	189.023
1.230.516	1.842.659	365.529
969.697	1.824.097	1.206.468
510.802	2.114.519	772.166
381.276	1.365.058	765.058
944.825	3009.17	1.736.851
62.523	370.791	245.058
775.46	1.538.568	883.56
1.064.764	3.301.188	726.506

BEAD INFLIX	BEAD ANTIGEN	BCG ANTIGEN INFLIX
0	0	0
0	0	0
0	0	0
0	1247.19	781.044
	15.712	6.086
0	208.343	114.783
85.585	1.449.388	233.202
0	400.584	77.147
150.663	169.143	43.998
34.998	138.108	34.986
34.833	350.667	0
0	0	0
0	0	0
0	0	0
0	0	0
0	0	0
102.362	89.32	23.06

BEAD INFLIX	BEAD ANTIGEN	BCG ANTIGEN INFLIX
37.953	799.83	461.769
57.981	81.063	38.868
87.906	66.897	19.167
0		
3.844		
10.24		
1.456	2.252	0
24.782		
0	0	0
6.054	5.866	0
23.086	15.206	0
8.07	5.978	0
442.82	6.008	0
121.316	283.797	0
5.104	13.53	9.582
5.222	0	0
178.32	181.464	79.64
293.512	307.36	181.464
120.288	328.4	102.624
128.288	767.848	78.08
63.472	566.392	46.712

37.953	799.83	461.769
120.288	328.4	102.624
293.512	307.36	181.464
25.104	113.53	39.582
63.472	566.392	246.712
0	36.236	0

**BEAD INFLIX**	**BEAD ANTIGEN**	**BCG ANTIGEN INFLIX**
123.915	93.942	70.737
62.997	94.005	54.393
105.54	30	14.856
367.245	117.639	25.62
62.811	31.848	0
68.829	0	0
92.751	59.082	40.926
52.539	51.465	29.802
77.238	77.238	43.002
0	0	0
0	0	0
0	0	0
127.062	71.247	104.446
	459.569	61.396
	9.92	511.144
0	0	0
0	0	0
120.288	328.144	226.368
65.432	187.776	91.024
73.184	712.072	146.416
65.896	136.024	46.712
167.992	188.632	80.328
98.56	114.616	88.08
55.184	111.912	62.616
123.915	93.942	70.737
65.432	187.776	91.024
367.245	1.117.639	825.62
562.997	1.294.005	854.393
68.829	358.369	258.398
997.236		
127.062	371.247	104.446
